# Process Evaluation and Costing of a Multifaceted Population-Wide Intervention to Reduce Salt Consumption in Fiji

**DOI:** 10.3390/nu10020155

**Published:** 2018-01-30

**Authors:** Jacqui Webster, Arti Pillay, Arleen Suku, Paayal Gohil, Joseph Alvin Santos, Jimaima Schultz, Jillian Wate, Kathy Trieu, Silvia Hope, Wendy Snowdon, Marj Moodie, Stephen Jan, Colin Bell

**Affiliations:** 1 The George Institute for Global Health, University of New South Wales, Sydney, NSW 2052, Australia; paayalgohil2@hotmail.com (P.G.); jsantos@georgeinstitute.org.au (J.A.S.); ktrieu@georgeinstitute.org.au (K.T.); sjan@georgeinstitute.org.au (S.J.); 2 School of Public Health, the University of Sydney, Sydney, NSW 2006, Australia; 3 Pacific Research Centre for the Prevention of Obesity and Noncommunicable Diseases (C-POND), Fiji National University, Nasinu, Fiji; arti.pillay@fnu.ac.fj (A.P.); arleen.sukhu@fnu.ac.fj (A.S.); jillian.wate@fnu.ac.fj (J.Wa.); 4 Independent Nutrition Consultant, Suva, Fiji; jimaima63@gmail.com; 5 Deakin Health Economics, Centre for Population Health Research, Faculty of Health, Deakin University, Burwood, VIC 3125, Australia; hope.silvia@gmail.com (S.H.); marj.moodie@deakin.edu.au (M.M.); 6 Global Obesity Centre, Deakin University, Geelong, VIC 3216, Australia; wendy.snowdon@deakin.edu.au (W.S.); colin.bell@deakin.edu.au (C.B.)

**Keywords:** evaluation, salt reduction, advocacy, public health policy, capacity building, costs, behavior change, food, nutrition, hypertension prevention

## Abstract

This paper reports the process evaluation and costing of a national salt reduction intervention in Fiji. The population-wide intervention included engaging food industry to reduce salt in foods, strategic health communication and a hospital program. The evaluation showed a 1.4 g/day drop in salt intake from the 11.7 g/day at baseline; however, this was not statistically significant. To better understand intervention implementation, we collated data to assess intervention fidelity, reach, context and costs. Government and management changes affected intervention implementation, meaning fidelity was relatively low. There was no active mechanism for ensuring food companies adhered to the voluntary salt reduction targets. Communication activities had wide reach but most activities were one-off, meaning the overall dose was low and impact on behavior limited. Intervention costs were moderate (FJD $277,410 or $0.31 per person) but the strategy relied on multi-sector action which was not fully operationalised. The cyclone also delayed monitoring and likely impacted the results. However, 73% of people surveyed had heard about the campaign and salt reduction policies have been mainstreamed into government programs. Longer-term monitoring of salt intake is planned through future surveys and lessons from this process evaluation will be used to inform future strategies in the Pacific Islands and globally.

## 1. Introduction

Recent analysis from the Global Burden of Disease study revealed that 3.7 million deaths per year could be attributed to consuming too much salt and that globally salt intakes are around 10 g/person/day, which is twice the World Health Organization (WHO) recommended maximum amount of 5 g/day [1]. Whilst an increasing number of countries are developing national salt reduction strategies [2], most are in the early stages of implementation and only a handful have demonstrated an impact to date [3]. Furthermore, the majority of experience to date comes from high income countries, so there is an urgent need to build the evidence about how to effectively implement programs in low and lower middle income countries [4]. The Fiji Sodium Impact Assessment Project (FSIA), funded by the National Health and Medical Research Council (NHMRC) as part of the Global Alliance for Chronic Diseases hypertension research program [5], evaluated the impact of multifaceted interventions to reduce population salt intake in Fiji and Samoa. 

**Figure 1:**
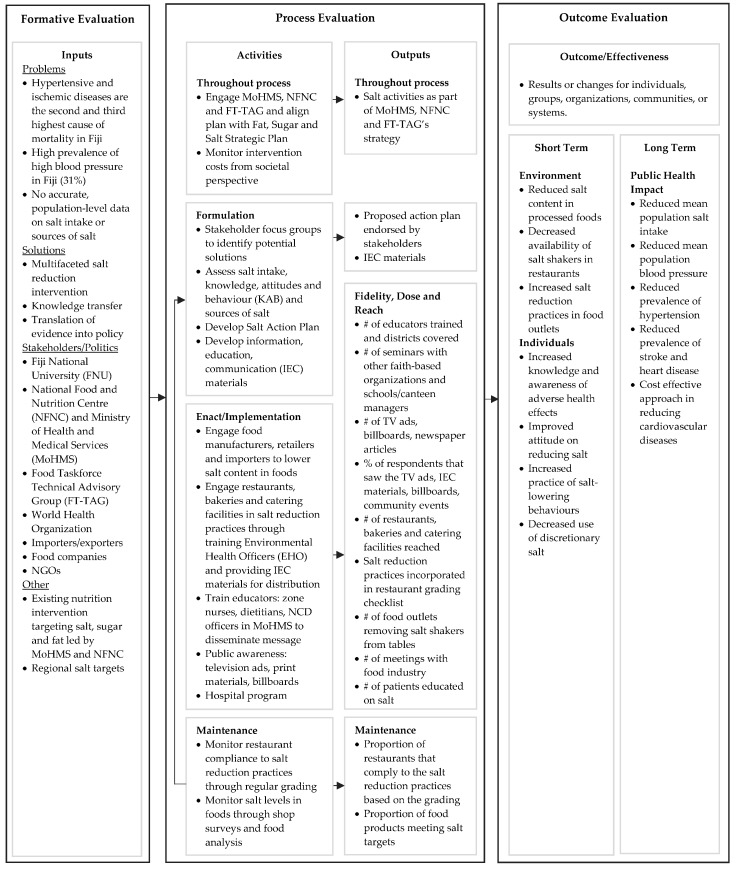
Centre for Training and Research translation logic model for Fiji Sodium Intervention Assessment Project (FSIA).

The intervention strategies were based on the WHO’s three pillars for creating an enabling environment for salt reduction [6], which is grounded in the theory that behavior change influencers span beyond education and information to include environmental and policy change [7]. The logic model for salt reduction programs (Figure 1) was informed by previous assessments of salt activities in the Pacific Islands [8]; baseline monitoring of salt intake, consumer knowledge attitudes and behaviors (KAB) related to salt and focus groups to understand stakeholder positions and barriers and opportunities for action in Fiji. The main causal assumption was that, given most salt consumed is already in processed foods and meals [9], reduction of salt levels in processed foods and meals would result in reduced salt intake. 

The resulting multifaceted intervention in Fiji targeted the whole national population, and had three strands: encouragement of the food industry to reduce sources of salt in the diet (through engagement of manufacturers and food service operators); strategic health communication (through targeted advocacy and a health educator training program); and a hospital program (education and reduction of salt in meals). The intervention was planned and implemented through collaboration between the Pacific Research Centre for the Prevention of Obesity and Noncommunicable Diseases (C-POND), the Fiji National Food and Nutrition Centre (NFNC) and the Wellness Unit in the Fiji Ministry of Health and Medical Services (MOHMS). It was overseen by a Food Taskforce Technical Advisory Group (FT-TAG) consisting of government, research and consumer organizations.

The impact of the population-wide intervention program was assessed through cross-sectional surveys of salt consumption patterns in a national sample at baseline and after 20 months [10]. The results of the impact evaluation were recently published in Nutrients [11]. The evaluation showed a 1.4 g/day drop in salt intake from a baseline of 11.7 g/day, however, this was not statistically significant. Lack of significant effect could have been due to the low response rates and small sample sizes obtained in the survey. However, limited intervention dose and duration is also a possible explanation. The outcome evaluation showed some improvements in consumer knowledge, as well as attitudes and behaviors regarding salt, but it was difficult to draw firm conclusions in view of the low response rates.

In order to get a better understanding of whether the lack of the effect on salt intake was due to the fact that the intervention didn’t work or whether it was because the intervention was not implemented effectively or over a long enough timescale, we conducted a comprehensive process evaluation. The results of this process evaluation will be used to inform future program implementation both in the Pacific Islands and globally. 

## 2. Materials and Methods

### 2.1. Research Questions

The process evaluation approach was informed by the Medical Research Council (MRC) framework and guidance for process evaluations of complex interventions [12] supplemented by a review of process evaluations of similar nutrition related interventions [13,14,15,16,17,18]. The first step was defining and understanding the causal assumptions underlying the intervention through the development of a logic model (Figure 1) and a detailed implementation plan during the project planning stages. We integrated the costing into the process evaluation as part of the routine monitoring.

The main questions for the process evaluation and costing were:

(1) Were the program interventions delivered with high fidelity, dose and reach?
(2) How did context affect implementation?
(3) What was the cost of different elements of the interventions?
(4) What lessons can inform continuation and/or replication of salt reduction strategies in other countries?

### 2.2. Data Collection

Data were collected through qualitative and quantitative measures integrated into the different stages of project implementation follows:

(1) Understanding the extent to which the intervention was actually implemented as planned (in line with the logic model and detailed implementation plan) through implementer self-report and collection of routine monitoring data supplemented through semi-structured interviews with key stakeholders (relevant government departments, consumer and health groups and food industry organizations that had been involved in the project).
(2) Understanding mechanisms of impact including whether specific groups were impacted differently through sub-analysis of the outcome data and routine monitoring data as well as semi-structured interviews with implementers and participants.

The semi-structured interviews were undertaken by the lead investigator who had a good understanding of the interventions without having been directly involved in program implementation. Interviews lasted approximately 40 min and were recorded. Additional questions were incorporated into the consumer, knowledge, attitudes and behavior survey at 20 months, as part of the intervention monitoring, to assess the extent to which people had been exposed to the campaign. 

### 2.3. Intervention Costing

A societal perspective was adopted for the costing, meaning that costs to participants, government and all sectors of the economy involved in the delivery of the intervention were included. The costing was done using pathway analysis to specify resources associated with the intervention strategies. Resource use and cost data were collected by the FSIA project team and the NFNC project officer in Fiji and analysed by the health economist team based at Deakin University, Australia.

The costs included:

(i) program-level expenses associated with the delivery and management of activities including transportation, accommodation, catering, venue hire, and administration;
(ii) costs associated with dissemination of information through radio, TV and newspaper;
(iii) costs associated with consultation with industry including group and one-to-one meetings and production of materials
(iv) human resource costs based on the hours involved for all individuals participating in any interventions and the relevant hourly wage rate.

The following costs were excluded from the analyses:

(i) research costs associated with intervention evaluation rather than implementation. The interventions were assumed to be operating in a ‘steady state’; therefore costs involved in set-up, research and development prior to the intervention were not included.

### 2.4. Data Analysis

Routine process monitoring data including activity logs, quarterly FSIA reports and annual reports were collated and tabulated according to the different intervention activities. The stakeholder interviews were transcribed verbatim. Each transcribed interview was individually imported to NVivo and the interviews were coded based on relevant themes in order to answer the process evaluation questions. 

Ethical Approval for this work was granted by the Human Research Ethics Council at the University of Sydney, Australia (15359), Deakin University, Australia (2013-020) and the Fiji National Research Ethics and Review Committee, Suva, Fiji (FNRERC 201307). 

## 3. Results

### 3.1. Data Sources

Routine monitoring data was collated from the costing spreadsheets, two FSIA annual reports, quarterly reports and reports on specific initiatives such as Salt Awareness Week 2014 and 2015. 

Table 1 provides an overview of the activities based on routine monitoring data with specific costs for interventions detailed in Section 3.4. Fifteen stakeholder interviews were conducted, with respondents comprising: three NFNC staff members, three Ministry of Health staff, three food industry stakeholders, three C-POND members, one WHO officer, one media representative, one hospital dietitian and one politician.

### 3.2. Overall Findings

Most people interviewed had been closely involved in the project and had a good understanding of what it was trying to achieve. The most consistent feedback from interviewees was that FSIA had been challenging to implement given time and resources. Reach was limited with routine monitoring data showing that most of the communication (except TV and radio) and industry meetings did not extend beyond Suva (population = 300,000 compared to 800,000 total population) and the hospital program was also only in the main hospital in Suva. Participants noted that, while there was strong awareness of the project, the strategic approach, in terms of both health communication and food industry engagement, could have been more clearly defined and communicated.

“*I think they really need to do that planning, the strategic planning to come out clear that okay this is a new program that has come in. This is what we need to achieve in the first year, second year, and third year. So these are the activities that we would expect.*”

Government workers and project staff said that the relatively short timescale (four years for baseline monitoring, interventions and post-intervention monitoring) meant that interventions were immature and had yet to take effect and that it needed to be continued.

“*So the time from planning to educating the educators and then getting them to get to the communities, being able to do something, getting information or us trying to go through the communities, the time frame was really short.*”

“*I don’t think we can expect to have achieved much given the timescale of the intervention. But, we now know how to do it and can continue.*”

Despite these challenges, some interviewees thought FSIA had helped to build research capacity and added value to existing efforts to improve the food supply.

“*There were already some interventions but the FSIA project really added value in terms of research which strengthened the case for further action. It also led to celebration of national salt week and stronger focus on salt through salt, fat and sugar strategy.*”

“*The project has helped us in relation to our NCD work and working with industry, but also in relation to research.*”

Whilst the results of the outcome evaluation were unknown at that stage, most interviewees, including those from government, research and industry, felt that the intervention strategies should be continued through integration into government programs. In fact, the recommendations from the project have already been used to inform the new Fiji Nutrition and Food Security Policy and Framework 2018–2022 and there are plans to repeat the monitoring of salt intake through the next WHO STEPwise approach to surveillance of noncommunicable disease risk factors (STEPS) survey (scheduled for 2018).

“*This is a great project. We now know what the nations’ salt intake is and we have structures and strategies in place to reduce it. It would be a shame now if it didn’t continue. The work needs to be incorporated into the Wellness Centre and the FT-TAG group needs to be mandated to oversee ongoing implementation.*”

#### 3.2.1. Project Governance

Project governance was seen as both a strength and a weakness of the project. Most people felt that it was appropriate that the research project was led by the research organization, C-POND and overseen by a multi-stakeholder advisory group. Also, there was strong recognition of the achievements and resilience of the C-POND team in effectively completing the project, particularly in view of the relatively short timescale and Cyclone Winston which affected post-intervention monitoring. Involvement of government was seen as a key strength of the project.

“*Certainly for Fiji I think the willingness of the Ministry of Health to be a full partner in this and to be involved throughout, I think has been absolutely key. Running this sort of intervention research project just from academia would be a huge problem and just be a mistake.*”

However clearer allocation of the roles of different agencies might also have supported more effective implementation of the industry strategy and led to greater reach for the communication of the health messages.

“*Task forces were set up, meetings were held, targets were agreed, IEC [Information, Education, Communication] materials were produced. But how far did the materials actually reach, who was responsible for making sure the industry even knew about let al.one adhered to the targets?*”

“*Would have been useful to have regular dissemination about the project—some sort of stakeholder newsletter, to keep people up to date and remind them of commitments, feedback on progress etc.*”

#### 3.2.2. Engaging the Food Industry to Reduce Salt in Foods and Meals

##### Food Business Operators

Based on the fact that most salt is in processed foods and meals a priority strategy was the get food businesses to reduce salt in foods and meals. However, interviewees indicated the strategy to engage companies was unclear. Whilst the voluntary targets for salt levels in foods had been agreed with industry in 2012, no mechanism was put in place to ensure compliance. The FT-TAG group was never formally established (so there was no clear Ministerial mandate). Also, the remit of the FT-TAG expanded to include fat and sugar reduction strategies so the focus on salt was less strong. At the same time, responsibility for the industry engagement work passed from C-POND to a WHO sponsored worker based at the NFNC during the intervention period. Many industry contact people changed, meaning some of the momentum was lost, which was a further barrier to effective engagement.

“*If they could just properly share with us the contacts… what are the commitments the food industry have mentioned to them. And for me to just follow up from there so there’s a continuity and a link between the two.*”

Previous effective salt reduction interventions with industry have relied on strong government leadership. However, stakeholders interviewed highlighted the political changes (elections leading to new Ministers), industry lobbing and lack of food technological expertise as additional challenges.

“*It’s the way our system is at the moment… Facing a big challenge. Particularly when you’re fighting against people like Coke and Nestle. Marketing is a big…*”

“*I really need somebody who can actually talk across to the food industry.*”

One food manufacturer interviewed said that most food companies were now fully aware of the importance of reducing salt in food products. Another company reported reformulating products in line with the voluntary targets that had been agreed prior to the project commencement. Most of the people interviewed reported that there appeared to be greater availability of “reduced salt” or “low salt” options in stores. However, one person indicated that companies were unclear about whether they should to act now or wait for government regulation. Several other stakeholders said that companies were not convinced about the health benefits of reformulating their foods, that they were not aware of any involvement of the Department of Trade and Industry and would not prioritize salt reduction in the absence of government regulation.

“*They were very honest in saying that until and unless it’s mandatory, we don’t see the priority to do this*”

“*The other big thing is voluntary. I have a feeling you need to regulate. But how to do it is the challenge…*”

##### Restaurant and Catering Facilities

The restaurant and catering sectors were engaged through the Environmental Health Officers (EHOs) (employed by the MoHMS) who were given two training session on salt reduction and provided with posters for distribution in restaurants/catering institutes. Taking salt shakers off the table was also integrated into MoHMS’s restaurant grading scheme which was communicated and enforced through EHOs. This was identified as a positive outcome of the project.

“*And one of the divisional environmental health officers from the northern division had advised us that they had removed salt from all their tables, as a rule in the northern division. So those were some outcomes of the intervention education that we had.*”

“*And there has been some visible changes. Most of the main restaurants we go to, they don’t give salt… It’s no longer on the table, you have to ask.*”

However, there was no follow-up activity to assess the extent to which the posters were distributed. The stakeholder interviews revealed that the Food Enforcement Unit in the MoHMS did not consider that it had a role in monitoring voluntary targets, and said the team would be unable to take on additional work without the provision of additional resources. Therefore no records were collected on how many restaurants had included salt reduction activities as part of the restaurant grading scheme.

#### 3.2.3. Health Communication: Targeted Advocacy and Training of Educators

##### Targeted Advocacy

The routine monitoring data (Table 1) showed that various communication materials (pamphlets, posters, booklets and DVDs) were produced and distributed fairly widely through events and educator training sessions. Faith-based organizations, The Ministry of Women, Children and Poverty Alleviation and Ministry of iTaukei affairs, are usually considered effective channels for disseminating information to communities in Fiji. However, neither the faith-based groups nor these two ministries were interested in engaging (citing other priorities) which limited the project reach. Instead, information was mainly disseminated through routine health promotion work of the NFNC and MoHMS and annually at national events. Two TV adverts were screened and there was media coverage on TV/radio to coincide with national events which means the messages were disseminated to regions beyond Suva. Based on the KAB survey post-intervention (*n* = 272), 73% of respondents reported that they had heard about the campaign from one or more sources. The most common source of exposure to the salt reduction campaign was through TV (32%), followed by health workers (27%), radio (26%), IEC materials (20%), community events (8%) and billboards (4%).

Those interviewed reported a much greater recognition of the importance of reducing salt in the diet amongst the general public as well as key government and health organizations However, some people expressed doubt about the reach of the campaign and reported the need to sustain efforts to so that this new knowledge could be translated to behavior change.

“*My concern is what percentage of the community we’ve reached regarding this information. Particularly the rural…*”

“*But people are being now more aware. I don’t know how much of it gets it into behavior change, but they do know and I have started talking about it in terms of groups when they’re getting along.*”

##### Training of Educators

Information was also disseminated through a training program for health educators covering Fiji’s 21 health districts. Seventy-five educators—zone nurses, dietitians, NCD project officers and health workers participated in workshops that covered the adverse effects of salt, using of salt substitutes for cooking and flavoring food, identification of reduced sodium products in similar categories of products by reading nutrient information panels and creation of demand for reduced salt products and meals. Twenty-seven per cent of the people who completed the KAB survey at the 20 month time point, said they were aware of the campaign through health workers. Interviewees thought the training sessions were informative but several people noted that there was no clear strategy or guidance for how the increased knowledge of the participants should then be disseminated at the community level.

“*FSIA (The project) should have worked more strategically with NFNC to get to the schools and through the Head of the NHS dietitians to get the messages to the community via the dietitians. And there needed to be a strategy—not just one-off communications.*”

#### 3.2.4. Salt Reduction in Hospitals

The Colonial War Memorial Hospital (CWMH), the main hospital in Fiji, located in Suva, made a commitment to reducing salt for staff and patients. Whilst some of this work to reduce the salt in patient meals was initiated prior to the FSIA project, activities reported during the project included training food service staff in the preparation of low salt meals, reducing salt in staff meals and taking salt shakers off the table and patient trays. The amount of salt available per person (hospital staff) per day from meals was reduced from 4.8 g in the second quarter of 2014 to 3.7 g in the same quarter of 2015. Dietary training covering the why and how of reducing salt was also provided for around 50 patients and relatives in the Special Outpatients Department. 

Unfortunately, due to limited time and resources, these activities were not replicated in hospitals in the other geographic divisions of the country although there were plans to replicate in other Divisions and extend to workplace catering guidelines thereafter. Materials were distributed widely to the sub-divisional hospitals but it is not clear to what extent they were used.

“*Because we have the lovely bags with the posters. … when I go through the sub-divisional hospitals, I don’t see them in the outpatients clinics where they sit around and wait… Hypertension and diabetes clinics… So I guess the extra step we should’ve taken was to ensure that we just go around again and make sure they have it up.*”

### 3.3. Sub-Analysis of Outcome Data

One of the recurring themes from the stakeholder interviews was the fact that Cyclone Winston had devastated much of the country during the 20 month follow-up monitoring which may have impacted the results. Sub-analysis of the main outcome data showed no differential impact of the intervention by age or ethnicity. However, there was a large and statistically significant reduction in salt intake of females in the Central division, compared to the other divisions. There was also a large and significant difference in salt intake between people in areas affected by the cyclone and those in areas not affected by the cyclone, with the cyclone areas consuming much more salt. Examination of reported exposure to the communication campaign (Table 2) showed that 73% of those surveyed had been exposed to the campaign by one or more sources—the most common source was TV, followed by health workers, radio and then posters/information materials. Exposure was fairly similar between the Central division and other divisions, for both males and females.

### 3.4. Intervention Costs

The overall cost of implementing the sodium reduction interventions in the specified time period was FJD $277,410. This equates to $0.31 per person based on the 2013 population of Fiji (881,065). Approximately half (49.5%) of the costs were related to human resources. Of the balance, the largest component was for printing of promotional materials (39.2%), with the largest expense being the purchase of TV adverts (33.9%) whilst meeting expenses (catering) accounted for 9.6% and travel expenses (1.7%) (Table 3).

## 4. Discussion

This is the first comprehensive process evaluation of a population-wide intervention to reduce salt. It demonstrated that the fidelity, dose and reach of FSIA was relatively low, partly due to the fact that the intervention was vulnerable to contextual issues such as poorly defined organizational roles, political changes, and natural disasters (Cyclone Winston). It also highlighted a timeframe that was insufficient for delivering the intervention with full fidelity. On the other hand, the process evaluation highlighted added value of the project in providing a robust estimate of how much salt people in Fiji are eating and the importance of reducing salt, strengthening research capacity and establishing mechanisms for engaging with the food industry to improve the food supply. The project also influenced government policy, with salt reduction activities now integrated into the work of the NFNC and MoHMS and plans to repeat monitoring of salt intake as part of the next WHO STEPS survey of NCD risk factors planned for 2018.

The likely impact of Cyclone Winston on the project was further highlighted through the process evaluation. Analysis of intervention exposure (Table 2) showed no differential exposure by division, thus challenging the theory that the significant effect in females in the Central division might be explained by the intervention dose being stronger there. The alternative explanation is that this was the only area where the monitoring took place before the cyclone hit in February 2016, and that the cyclone, which delayed the monitoring and affected diets due to the provision of rations, might have negated any potential impact of the intervention. Whilst the small sample sizes, meaning that the main outcome analysis was likely underpowered, make it impossible to draw firm conclusions, it is highly plausible that the cyclone had some sort of impact on the results.

Routine monitoring data showed the distribution of resources was fairly even, meaning the reach of the communications was wide. However, there was no data to show the extent to which resources were used and participant feedback suggested dissemination strategies were unclear. Likewise, whilst 73% of the population were exposed to the campaign, only a third (33%) and just over a quarter were exposed to TV or radio respectively and it is not clear how many times. The CDC recommends that 75–85% of the target audience need to be exposed each quarter of the year and that a campaign should run at least three to six months to achieve awareness of the issue, six to 12 months to influence attitudes, and 12 to 18 months to influence behavior [19]. A recent comprehensive review of salt reduction communication campaigns also suggested that behavior change programs on salt are more likely to be effective if grounded in a theoretical framework [20]. Future communication strategies need to identify and target specific salt related behaviors to change and develop clear strategies for broader dissemination of behavior change communications over an extended time frame to increase the likelihood of effectiveness [21].

Complex interventions to reduce salt include policy changes which require adequate time and strong governance to implement. The importance of establishing robust and transparent mechanisms for engaging and monitoring industry in relation to salt targets has been highlighted by previous research [22]. Whilst the agreement on voluntary targets to reduce salt intake was established in Fiji, strategies to ensure these targets were adhered to, were not effectively implemented during the timescale of the research project. Considering the large contribution of processed foods to salt in the diet, this is likely one of the main reasons for the lack of intervention impact and emphasises the importance of strengthening policy implementation, potentially through the use of legislation, and ensuring adequate time for interventions to take effect as part of future salt reduction efforts.

Lack of effective governance mechanism to implement and monitor nutrition polices in Low and Middle Income Countries (LMICs) has been identified as an ongoing challenge in previous research [23,24,25]. The salt reduction research project was led by C-POND, a regional research organization working on NCDs. It was intended that the intervention be implemented primarily through the MoHMS and the NFNC. However, the roles of the NFNC and MOHMS in implementing the program were never clearly defined and much of the salt reduction intervention effort was left to C-POND, which had limited financial resources (the total cost of the intervention was FJD227.410 including personnel time). 

The process evaluation has identified a range of lessons that will be useful in informing future salt reduction interventions, both in the Pacific Islands and globally. Firstly, four years is not long enough to develop, implement and evaluate a project. Whilst this project did not show what time frame is required to impact on dietary sodium levels at a population level, other countries have demonstrated an impact after five years [26]. Secondly, adequate resources need to be provided to implement and monitor program impact. The cost of this intervention was moderate resulting in a relatively low intervention dose. Likewise, greater resources for monitoring could have helped to increase sample size, increasing the power of the study to detect change. Thirdly, strong multisectoral governance mechanisms need to be established for implementation and monitoring of policies. Strong government leadership is preferable, particularly to ensure that the food industry adheres to agreements to reduce salt in processed foods and meals. Health ministries often have established mechanisms for communicating to communities and undertaking health surveillance that salt reduction interventions need to harness. Ministries of trade and industry and education should also be involved in planning and implementation. Fourthly, there needs to be a clear strategic approach to communication activities to change behavior with adequate replication. Also, whilst policy initiatives are usually best implemented at a national level, limited resources and the challenges of reaching all areas means communication campaigns might be better piloted in certain regions before being rolled out nationally. Lastly, communication with key stakeholders to ensure that everyone is clear about the objectives and approach at different stages throughout the project is important. Maintaining an up-to-date stakeholder database can help facilitate this. Training to support project implementation, including food industry engagement, needs to be repeated regularly, particularly when new project staff are engaged. 

Many of these lessons are relevant to other implementation science projects and some have been highlighted in previous process evaluations of food-related interventions in the region. [16,17,18]. Common to all these interventions is that they were being implemented in the “real world” working through government institutions where it is harder to control processes as part of a research project. On the other hand, increased research capacity and mainstreaming of programs into government policies and practices increases the likelihood of programs being sustained and longer-term benefits being realized.

### Strengths and Weaknesses

Strengths of this process evaluation include the mapping of causal assumptions at the start of the intervention, transparency of the process and the openness and willingness of stakeholders, including government and industry organizations, to discuss challenges related to the project. A weakness was the fact that the main outcome study was underpowered which made it difficult to draw firm conclusions on many of the issues. The limited number of interviews, particularly with representatives from industry, may have biased the views obtained. Also, the multiple players involved in the intervention meant that routine monitoring data was not collected effectively by all parties so it was not always possible to quantify the extent of the activities, making it difficult to determine dose and reach as well as to assess the cost of some activities. The costing data is fairly comprehensive and indicates a low-cost intervention, but does not capture the pre- and post-intervention monitoring, which is a challenging and expensive element of all salt reduction interventions [27].

Whilst an extensive sub-analysis of the main outcome data was undertaken, only some of the issues are reported here and the low response rates means that the sub-analysis was also likely underpowered.

## 5. Conclusions

This process evaluation demonstrates the impact of the project in terms of policy outputs and increased research capacity in Fiji and has identified lessons to inform ongoing salt reduction efforts in the Pacific Islands and globally. 

## Figures and Tables

**Table 1:** Summary of salt reduction intervention activities in Fij from routine monitoring data.

Target Group and Objective	Summary of Intervention Activity	Numbers	Distribution
**Consumers and stakeholders** To raise awareness of dangers of too much salt, recommended level of salt consumption, and hidden salt levels of food	C-POND communication and advocacy materials (pamphlet, posters, booklets, DVD) IEC materials distributed through nurses and dietitians and the NFNC and Wellness Centre Information also available on NFNC and MoHMS websites TV adverts, regular print media coverage and C-POND newsletter Salt awareness week (SAW) events	681 Pamphlets 731 Sets of 3 posters 200 Salt facts booklets 32 Salt The Hidden Danger Digital Video Disk (DVDs) 2 TV adverts distributed free to air Media coverage on salt 4–5 times per year 2 SAW events 1 Motorway Billboard	- Materials distributed annually at national events such as Salt Week, Health Day, Nutrition Month, Hibiscus Festival, Noncommunicable disease (NCD) Month and World Food Day (mainly in Central Division) - Regular coverage through TV, radio and print media and information available on MoHMS website (available throughout the regions)
**Health educators** To provide training and resources so people already working in the community can integrate salt reduction into their programs	Training of educators (nurses, dietitians, red cross workers etc) on salt reduction Other government Ministries, faith based organizations and nutrition educators were consulted PEN Model Training included a module on salt reduction Presentation to Heads of Schools and Canteen Managers Attempts to facilitate training and dissemination of materials through Ministry of Women, Ministry of Youth and Ministry of Education	75 educators trained, August–September 2014 Meetings held with 9 organizations but only one group—Muslim Markaz Women’s Group went on to deliver training (41 participants) Pen training 7 groups October–December 2015 100 participants in schools and canteen manager training, August 2014	- Dieticians and nurses trained covered all 21 districts - Additional one-off trainings took place in Central Unit and Western Unit - Training for MoHMS Food Unit workers who cover all districts - Muslim Women’s Groups in all districts - School Canteen managers in all districts
**Food business operators** To encourage and support manufacturers and retailers to produce and sell lower salt Foods	Promotion of voluntary targets for salt levels in foods agreed between government and food industry Food industry consultation meetings held by C-POND Nutrition consultant/WHO worker hired to support industry negotiations FT-TAG group set up and met monthly to advise on progress and to develop salt, fat and sugar reduction strategy Category specific Food and Beverage Health action groups established One to one meetings with Food companies (*n* = 9): restaurants and takeaways (*n* = 3) and retailers (*n* = 3)	Targets agreed September 2012 Cross cutting industry consultation meetings held in September 2013 and July 2014 C-POND held 15 meetings with industry organizations in 2014 6 Category specific groups established No records of meetings with these groups.	- All industry meetings in Vitu Levi where companies operate.
**Restaurants and catering facilities** To raise awareness of the importance of reducing salt and provide incentives for taking action to reduce salt for restaurant and catering staff	Posters distributed to the Environmental Health Officers for distribution in restaurants/catering institutes. Taking salt shakers off the table integrated into MoHMS restaurant grading scheme	100 posters distributed, November 2014–2015 No data on number of restaurants graded	- No record on numbers of restaurants graded
**Hospitals** To educate hospital staff and dieticians about the importance of reducing salt in diet so that they might introduce salt reduction initiatives into hospitals	Salt reduced in hospital meals (from 4.8 g/head per day (2014) to 3.7 g/head/day) Salt shakers have been removed from the staff dining room Food Service staff trained on preparation of low salt meals Education of patients and relatives through hospital dietitians	1 hospital in Suva, 2015 No numbers available on staff trained 50 patients educated	- Suva only

Notes: C-POND—Pacific Research Centre for the Prevention of Obesity and Noncommunicable Diseases; IEC—Information, Education and Communication; PEN—Package of Essential NCD interventions; MoHMS—Ministry of Health and Medical Service; NFNC—National Food and Nutrition Centre; WHO—World Health Organization; FT-TAG: Food Taskforce Technical Advisory Group.

**Table 2:** Percentage of respondents who heard about the campaign by sex and division.

		Any Source	Radio	TV	Bill Boards	Posters, Information Materials	Community Event	Health Worker	Others
Total Sample	Overall	72.7	25.9	32.0	3.5	20.4	8.1	26.5	1.6
	Male	67.3	23.4	27.0	3.5	13.7	8.9	20.6	0.8
	Female	78.3	28.4	37.1	3.5	27.3	7.2	32.6	2.3
Central Division	Overall	69.1	18.1	30.0	3.2	14.9	5.3	21.1	1.0
	Male	62.5	16.7	29.7	2.1	8.2	9.8	15.2	2.1
	Female	75.3	19.4	30.2	4.3	21.2	1.1	26.6	0.0
Other Division	Overall	75.3	31.2	33.3	3.7	24.1	9.9	30.3	1.9
	Male	70.4	27.6	25.3	4.3	17.1	8.3	24.0	0.0
	Female	80.6	35.2	42.1	2.9	31.9	11.8	37.1	4.0

**Table 3:** Intervention costs.

**(a) Breakdown of Total Costs by Resource Type (FJD)**			
Type of Cost	Total Cost	% Consumable Costs	% Total Costs
Personnel	$138,506 *		49.93%
Promotional materials	$106,685	76.80%	38.46%
Meeting expenses (catering)	$16,001	11.52%	5.77%
Travel	$16,219	11.68%	5.85%
Total costs	$277,410	100.00%	100.00%
**(b) Breakdown of Total Costs by Activity Type (FJD)**			
Activity Type	Cost		% Total Costs
Consultation (meetings, focus groups)	$49,757		17.94%
Salt Awareness weeks	$9801		3.53%
Training	$8030		2.89%
TV adverts	$94,226		33.97%
Activities across all above	$115,596		41.67%
	$277,410		100.00%

* Participant time was costed as the average wage in Fiji. For persons involved in a professional capacity, an average hourly rate (based on 45-h week) was calculated based on average annual salaries according to different salary levels within each profession. It was assumed that the FSIA Research Fellow and Research assistant spent 50% of their time over the 20 months intervention period on intervention delivery activities.

## Abbreviations

C-POND	Pacific Research Centre for the Prevention of Obesity and Noncommunicable Diseases
EHO	Environmental Health Officer
FNU	Fiji National University
FSIA	Fiji Sodium Impact Assessment Project
FT-TAG	Food Taskforce Technical Advisory Group
GACD	Global Alliance for Chronic Diseases
IEC	Information, Education and Communication
KAB	Knowledge, attitudes and behaviors
LMIC	Low and Middle Income Countries
MoHMS	Fiji Ministry of Health and Medical Services
MRC	Medical Research Council
NCD	Noncommunicable diseases
NFNC	National Food and Nutrition Centre
NHMRC	National Health and Medical Research Council of Australia
NNS	National Nutrition Survey
PEN	Package of Essential NCD interventions
SAW	Salt Awareness Week
STEPS	WHO STEPwise approach to surveillance of noncommunicable disease risk factors
WHO	World Health Organization
